# Cross-Serotype Reactivity of ELISAs Used to Detect Antibodies to the Structural Proteins of Foot-and-Mouth Disease Virus

**DOI:** 10.3390/v14071495

**Published:** 2022-07-08

**Authors:** Anna B. Ludi, Alison Morris, Simon Gubbins, Amin Asfor, Madeeha Afzal, Clare F. Browning, Santina Grazioli, Efrem Alessandro Foglia, Ginette Wilsden, Alison Burman, Emiliana Brocchi, David J. Paton, Donald P. King

**Affiliations:** 1 The Pirbright Institute, Ash Road, Pirbright, Woking GU24 ONF, UK; anna.ludi@pirbright.ac.uk (A.B.L.); alisonrand880@hotmail.com (A.M.); simon.gubbins@pirbright.ac.uk (S.G.); amin.asfor@pirbright.ac.uk (A.A.); madeeha.afzal@pirbright.ac.uk (M.A.); clare.browning@pirbright.ac.uk (C.F.B.); ginette.wilsden@pirbright.ac.uk (G.W.); alison.burman@pirbright.ac.uk (A.B.); donald.king@pirbright.ac.uk (D.P.K.); 2 Istituto Zooprofilattico Sperimentale della Lombardia e dell’Emilia Romagna (IZSLER), Via Bianchi 9, 24125 Brescia, Italy; santina.grazioli@izsler.it (S.G.); e.foglia@izsler.it (E.A.F.); emiliana.brocchi@gmail.com (E.B.)

**Keywords:** foot-and-mouth disease virus, serology, cross-reactivity, antibody, SP-ELISA

## Abstract

Antibodies to the foot-and-mouth disease virus (FMDV) capsid induced by infection or vaccination can provide serotype-specific protection and be measured using virus neutralization tests and viral structural-protein (SP-)ELISAs. Separate tests are needed for each serotype, but cross-serotype reactions complicate serotyping. In this study, inter-serotypic responses were quantified for five SP-ELISA formats by testing 294 monovalent mainly bovine sera collected following infection, vaccination, or vaccination and infection with one of five serotypes of FMDV. Over half of the samples, representing all three immunization categories, scored positive for at least one heterologous serotype and some scored positive for all serotypes tested. A comparative approach to identifying the strongest reaction amongst serotypes O, A and Asia 1 improved the accuracy of serotyping to 73–100% depending on the serotype and test system, but this method will be undermined where animals have been infected and/or vaccinated with multiple FMDV serotypes. Preliminary studies with stabilized recombinant capsid antigens of serotypes O and A that do not expose internal epitopes showed reduced cross-reactivity, supporting the hypothesis that capsid integrity can affect the serotype-specificity of the SP-ELISAs. The residual cross-reactivity associated with capsid surface epitopes was consistent with the evidence of cross-serotype virus neutralization.

## 1. Introduction

Foot-and-mouth disease (FMD) is an acute, highly contagious disease that affects cloven-hoofed domestic and free-living mammals in many parts of the world with major impacts on farming and the trade of livestock and their products. The causative virus (FMDV) is unenveloped with an RNA genome and is an Aphthovirus in the Picornaviridae family. The FMDV capsid is composed of four structural proteins (VP1–4) that are assembled into roughly triangular-shaped protomeric subunits. Five protomers form a pentamer and 12 pentamers form the capsid [1,2]. Seven immunologically distinct serotypes of the virus have been described, but serotype C appears to be extinct [3], and the three Southern African types (SATs) are mainly confined to Africa.

FMD control is supported by serological assessments of the presence and prevalence of FMDV infection, and immunity checks of the quality and antigenic relevance of vaccines, and of the responses of livestock populations to vaccination programs. There are two main categories of tests used for FMD serology [4]: (i) tests that detect non-protective antibodies to non-structural viral proteins (NSPs) that are conserved between serotypes and induced by infection but not elicited by purified vaccines, and (ii) tests for antibodies targeted at the structural proteins (SPs) of the capsid that include potentially protective and serotype-specific surface epitopes on VP1–3. Internal capsid epitopes, including those on VP4, are more conserved between serotypes and the antibodies against them have not been shown to be protective [5]. Virus capsids are the principal antigenic component of FMD vaccines and are derived from inactivated cell-culture-grown viruses. After vaccination or infection, animals may develop antibodies to both external and internal capsid epitopes and tests for antibodies for these SPs are used as indicators of past infection or vaccination and to estimate immune protection. The World Organisation for Animal Health (OIE), Paris, France, considers the virus neutralization test (VNT) to be the “gold standard” for the detection of serotype-discriminating antibodies against the FMDV SP and as a predictor of protection [4]. Presumably, this test detects antibodies to external epitopes on the capsid surface. However, the VNT is slow and dependent on cell cultures and manipulation of the live virus; therefore, in-house and commercial serological ELISAs are more widely used to assess the immune status of animals in the field. Both the VNT and the liquid-phase-blocking ELISA (LPBE, an SP antibody test) were validated against vaccine potency tests as serological indicators of probable protection after vaccination [6,7]. Both are also used to estimate the antigenic match between vaccine and field strains of FMDV [4]. Like vaccines, SP-ELISAs use antigens derived from cell-culture-grown and inactivated capsids. As the FMDV capsid readily dissociates, for example, if vaccine cold chains are not strictly maintained, it is likely that the antigen presented to the host following infection or vaccination, as well as that on ELISA plates, will be a mixture of intact capsids on which only external surface epitopes are exposed and viral subunits presenting external and internal capsid epitopes. This will give rise to a variable ratio of serotype-specific and protective antibodies on the one hand and serotype-independent and non-protective antibodies on the other hand [8,9]. To enhance their stability and to reduce their susceptibility to heat and pH changes, recombinant empty viral capsids were engineered as putative vaccines with stabilizing mutations at the capsid subunit interphases [10].

Serological cross-reactivity between serotypes has long been recognized [11,12] and care is therefore needed in interpreting serological data, particularly for FMD surveillance studies in endemic countries where multiple serotypes circulate. However, the nature and extent of cross-reactivity with current serological ELISA platforms have not been confirmed. In this study, monovalent antisera collected from experimental animals immunized via vaccination and/or infection with five FMDV serotypes (O, A, Asia 1, SAT 1 and SAT 2) were assessed using five SP-ELISA systems, each comprising tests for 3–5 of these serotypes. To assess the cross-reactivity, the data were analyzed directly and by fitting multivariate normal distributions to the results for each animal using Bayesian methods.

The results showed that although sera are most likely to be positive when tested with an ELISA against the homologous serotype, there is still an appreciable probability (>10%) that a sample will be positive when tested with an ELISA against a different serotype. A preliminary comparison showed that the stabilization of ELISA antigens reduced cross-serotype reactivity of sera from animals immunized with serotypes O and A.

## 2. Materials and Methods

### 2.1. Serum Samples

Two hundred and ninety-four sera were selected from animals immunized with one of five serotypes of FMDV (O, A, Asia 1, SAT 1 and SAT 2), including a variety of topotypes and strains. The sera were collected from animals infected and/or vaccinated experimentally with a single FMDV serotype (i.e., monovalent). Similar numbers of samples came from infected, vaccinated and vaccinated-then-infected animals (94, 94 and 106, respectively). Most samples were collected from cattle (*n* = 261) infected with and/or vaccinated against serotypes O, A or Asia 1 at approximately 30 days after vaccination and/or infection. There were also 26 samples from sheep and 7 from pigs. Further details are shown in Supplementary Materials Table S1. The sera were stored at −20 °C for an average of 12.8 years (2.4–56 years).

### 2.2. ELISA Testing for Antibodies to FMDV Structural Proteins

The sera were tested for the presence of antibodies against the SP of three to five FMDV serotypes using five competitive ELISA systems. In each system, the same format but different specific antigen and antibody reagents were used for each serotype. These tests included two in-house polyclonal-antibody-based ELISAs from the FMD World Reference Laboratory (WRLFMD), Pirbright, as well as three widely used, commercially available kits that employ competitive monoclonal antibodies (mAbs) (Supplementary Materials Table S2). All these tests used cell-culture-derived and subsequently inactivated viral antigens. In each ELISA, the sera were tested singly at four dilutions in a threefold dilution series that started with the specified dilution recommended in each of the kits for a spot test (Supplementary Materials Table S3). For each dilution, a percent inhibition (PI) was calculated with reference to a negative serum, except for the IDVet ELISA, where the results are expressed in an inverse manner. For ease of comparison, IDVet results are shown as 100 × (1 − S/N), which corresponds to a PI, as used for other ELISA systems. The cut-offs used were as specified by the tests’ protocols (Supplementary Materials Table S3). An inconclusive range was stipulated for some tests, but for simplicity, inconclusive positives were scored as negative. End-point titers were not calculated, as many sera remained positive at the final dilution used.

### 2.3. In-House Liquid-Phase Blocking ELISA (LPBE) and Solid-Phase Competition ELISA (SPCE)

The antigens used in the tests, as well as the polyclonal capture and competitor antisera, were derived from the following FMDV strains: O Manisa, A22 IRQ 24/64, Asia 1 Shamir, SAT 1 RHO 12/78 and SAT 2 Eritrea. The LPBE [12,13] and the SPCE [14,15] use polyclonal rabbit antisera to capture SP antigens on ELISA plates, whilst polyclonal guinea pig antisera compete with test serum antibodies. For the LPBE, the antigen and test sera were incubated together before being captured on the plate; meanwhile, for the SPCE, the test serum competed with the competitor (guinea pig) antiserum for the antigen after capturing it on the plate.

The LPBE was carried out as described in [12], except that the blocking buffer consisted of 0.01 M phosphate-buffered saline containing 5% skimmed milk powder (Marvel) and 0.05% Tween 20. The guinea pig antiserum was pre-blocked with normal, non-immune bovine serum (Gibco, Waltham, MA, USA) at a ratio of 1:1.

The SPCE was performed as described [15], except that the guinea pig antiserum was pre-blocked with non-immune bovine serum (Gibco, Waltham, MA, USA) at a ratio of 1:1. The ELISA blocking buffer consisted of 0.01 M phosphate-buffered saline containing 0.05% Tween 20, 10% normal bovine serum and 5% normal rabbit serum.

For both assays, optical density values were obtained at 492 nm using an ELISA reader. Internal controls were included on every plate to quality control the testing procedure. These controls were previously calibrated in the assay and were compared and accepted against a running mean to ensure that the acceptance criteria of the assays were met.

### 2.4. Commercial ELISA Kits

Commercially available, serotype-specific FMDV antibody detection ELISA kits manufactured by Istituto Zooprofilatico Sperimentale, Brescia, Italy (IZSLER), PrioCHECK^®^ (Thermo Fisher Scientific Prionics AG, Waltham, MA, USA), and the ID Screen^®^ FMD commercial ELISA kits (ID.VET Innovative Diagnostics) were used according to the manufacturers’ instructions (except for the PrioCHECK and IDVet kits, where titration is not part of the manufacturer’s protocol). The IDVet and PrioCHECK kits covered three serotypes (O, A, Asia 1), whilst the IZSLER kits covered five serotypes (O, A, Asia 1, SAT 1, SAT2). The exact nature of the antigens and antibodies used in the kits are proprietary information, but all are solid-phase competition ELISAs in which test sera are added to ELISA plates pre-sensitized with capsid antigens, either directly coated or immune-captured depending on the manufacturers, and free epitopes are disclosed with a peroxidase-conjugated mAb.

### 2.5. Integrin-Capture ELISA

To study the contribution of external and internal capsid epitopes to cross-serotype reactivity, the impact of capsid dissociation on antibody binding was assessed with 12 bovine sera representing 2 naïve cattle and cattle immunized with serotypes O (*n* = 6) and A (*n* = 4), including SP-ELISA cross-reactive and non-cross-reactive examples. The ELISA system incorporated a recombinant alphaV beta6 integrin as a ligand for capturing the SP antigens of all serotypes [16,17,18]. The antigens were derived from FMDV strains O Manisa and A22 by expressing recombinant virus-like particles (VLPs) with a vaccinia virus system [10,19]. The VLPs were derived from either the sequence of a wild-type virus or included mutations to improve capsid stability. The VLPs were purified through a sucrose cushion and sucrose density centrifugation and the fractions containing purified VLPs were stored at 4 °C. The wild-type virus capsid antigens were tested with and without prior heat treatment at 60 °C for 10 min to dissociate the capsids.

A 50 mM carbonate buffer with pH 9.4 was used for coating the plates with 1 µg/mL integrin. PBS with 0.1% Tween 20 was used for washing, whilst PBS with 5% skimmed milk and 1% horse serum was used for blocking plates and for preparing serum dilutions. The binding of serum antibodies was detected with HRP-conjugated anti-bovine immunoglobulins. Incubation steps lasted 1 h at room temperature. After the final wash, bound antibody conjugate was revealed via a 10 min incubation with TMB and stopped using 0.6 M sulphuric acid. The absorbance was read at 450 nm and the results were expressed as the net optical density of each sample with or without an antigen.

The integrity of capsids in the antigen preparations was measured with mAbs. One of these (5B2) recognizes an internal epitope on VP2 that is common to all serotypes [20] and can only be bound if capsids are dissociated. The other two are neutralizing and specific for epitopes on the external surface of the capsid that are available in both intact and dissociated particles. One (D9; [21]) binds to the VP1 G-H loop of serotype O and the other (5F6; [22]) to a conformational epitope of serotype A. These were substituted for bovine serum samples in the integrin ELISA and detected with an HRP conjugated anti-murine antibody.

### 2.6. Virus Neutralization Test

Sixty sera from cattle immunized with serotypes O (*n* = 30) and A (*n* = 30), including mostly highly cross-reactive sera, and those evaluated in the integrin-capture ELISA were tested in a VNT at final doubling dilutions from 1 in 8 to 1 in 1024 against A22 IRQ 24/64 and O Manisa viruses [4]. These strains were homologous to the vaccine and/or challenge viruses against which the sera of the same serotype was raised for 80% (serotype O) and 30% (serotype A) of the samples. A reciprocal titer of 45 or more was considered positive, whilst titers less than 16 may be considered negative, depending upon the specificity required [4].

### 2.7. Analysis of Cross-Reactivity between ELISA Systems

The results were analyzed as two datasets, with the first involving antisera and tests for serotypes O, A and Asia 1, where all combinations of homologous and heterologous testing were complete. These data were analyzed to determine the sensitivity of homologous serotype detection and to see how comparative interpretations of qualitative and quantitative reactivities at different dilutions affected the test specificity.

The second dataset included the SAT 1 and SAT 2 serotype sera and tests, where it was not possible to test all of the sera against all five serotypes. The sample results for the five serotypes were analyzed using an approach that allowed for the fact that each serum sample was taken from an animal immunized with a particular serotype and was tested against multiple serotypes (so that the responses for each serotype may be correlated). To make the results comparable in a homogenized presentation, the IDVet results were shown as 100 × (1 − S/N), which corresponds to a PI, as is used for other ELISA systems. In addition, the methods enabled us to assess the impact of the immune status (i.e., whether the animal had been infected, vaccinated, or vaccinated and infected), species (i.e., whether the sample was bovine, ovine or porcine), duration of storage, and the time of sampling post-vaccination or -infection. The time of sampling for vaccinated/infected animals was typically given as days post-challenge. In most cases, the number of days post-vaccination when the challenge occurred was also known, but where it was not, animals were assumed to have been vaccinated 21 days before the challenge.

The response for serum from an animal immunized with FMDV serotype j and tested using an ELISA against different serotypes was modeled using a multivariate normal (MVN) distribution, with the responses being logit transformed to improve the normality of the data. Specifically, the responses for the serum taken from animal *j* when tested using an ELISA for each serotype (**p***_j_* = {*p_jk_*}; subscript *k* denotes the test serotype) was given as

(1) $$p_{j}\sim \mathrm{MVN}(\mu_{j},\Sigma ).$$

where

(2) $$p_{jk}=\log\left(\frac{PI_{jk}-\underset{j}{\min}(PI_{jk})}{\underset{j}{\max}(PI_{jk})-PI_{jk}}\right),$$

and *PI_jk_* is the observed response against serotype *k* for animal *j*. In Equation (1), **μ***_j_* = {*μ_jk_*} is a vector of expected transformed responses for the animal against each serotype such that

(3) $$\mu_{jk}=\alpha_{SERO_{j},k}+\beta_{INF_{j}}+\gamma_{SPP_{j}}+\delta t_{j}+\varepsilon a_{j},$$

where *SERO_j_*, *INF_j_* and *SPP_j_* are the serotype with which the animal was infected or vaccinated, the immunization status (infected, vaccinated, or vaccinated and infected) and the species (bovine, ovine or porcine) for animal *j*, respectively, and **Σ** is the variance–covariance matrix. In Equation (2), *α_jk_* is the expected transformed response for a bovine infected with FMDV serotype *j* when tested with an ELISA against serotype *k*, *β_i_* is the effect of infection status *i* on the response (i.e., the difference in expected response between a vaccinated or a vaccinated and infected animal and an infected one), *γ_s_* is the effect of species *s* on the response (i.e., the difference in expected response between a sheep or a pig and a bovine animal), *t_j_* is the time at which the sample was taken (in days after the earlier of infection or vaccination) and *a_j_* is the age of the sample (in days).

The results for each ELISA were analyzed independently. Parameters were estimated in a Bayesian framework using OpenBUGS (version 3.2.3; https://www.mrc-bsu.cam.ac.uk/software/bugs/openbugs/ (accessed on 6 July 2022)). Weak priors were assumed for all parameters: normal (with mean 0 and variance 100) for the *α_jk_*s, the *β_i_*s, the *γ_s_*s, *δ* and *ε*; Wishart (with a diagonal matrix with elements 0.001 for the scale matrix and *n* equal to the number of serotypes for the degrees of freedom) was assumed for the precision matrix (i.e., the inverse of the variance–covariance matrix). Two chains, each of 11,000 iterations, were run with the first 1000 iterations discarded to allow for burn-in of the chains. Convergence was monitored visually and using the Gelman–Rubin statistic implemented in OpenBUGS. The evidence for an influence on the expected response of each of the factors in Equation (2) (i.e., immunization status, species, time of sampling and age of sample) was assessed by comparing the deviance information criterion (DIC) for models in which terms were removed from the model [23].

The practical implications of the results were explored by using the fitted model to estimate the percentage of samples that were expected to be positive when sera from animals immunized with a given serotype were tested with a serotype-specific ELISA (possibly against a different serotype from the immunized animals). Specifically, samples were drawn from the joint posterior distribution for the parameters, and for each sampled parameter set, the model in Equation (1) was used to generate a simulated set of responses, and hence, the percentage of positive samples (based on the specified cut-off for the test used). For each combination of immunizing serotype and test, 1000 samples were drawn from the posterior distribution and the responses that were simulated for one, ten, one hundred or one thousand animals.

## 3. Results

### 3.1. Homologous Testing

**Figure 1:**
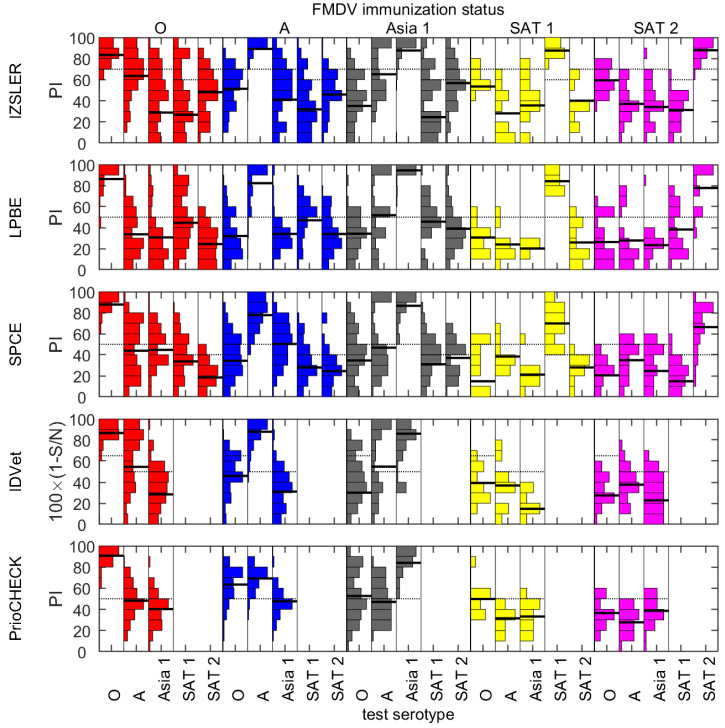
Observed responses for sera from animals immunized with FMDV serotype O (red), A (blue), Asia 1 (grey), SAT 1 (yellow) or SAT 2 (magenta) (indicated above the top panel) when spot tested using an ELISA against FMDV serotype O, A, Asia 1, SAT 1 or SAT 2 (indicated below the bottom panel). Results are shown for spot tests with five SP-ELISAs: IZSLER, liquid-phase blocking ELISA (LPBE), solid-phase competition ELISA (SPCE), IDVet and PrioCHECK (note: for IDVet and PrioCHECK™, tests were not available against serotypes SAT 1 or SAT 2). Each plot shows the observed responses (bars), the mean response (solid black line) and the cut-off for classifying a sample as positive (black dotted line).

**Figure 2:**
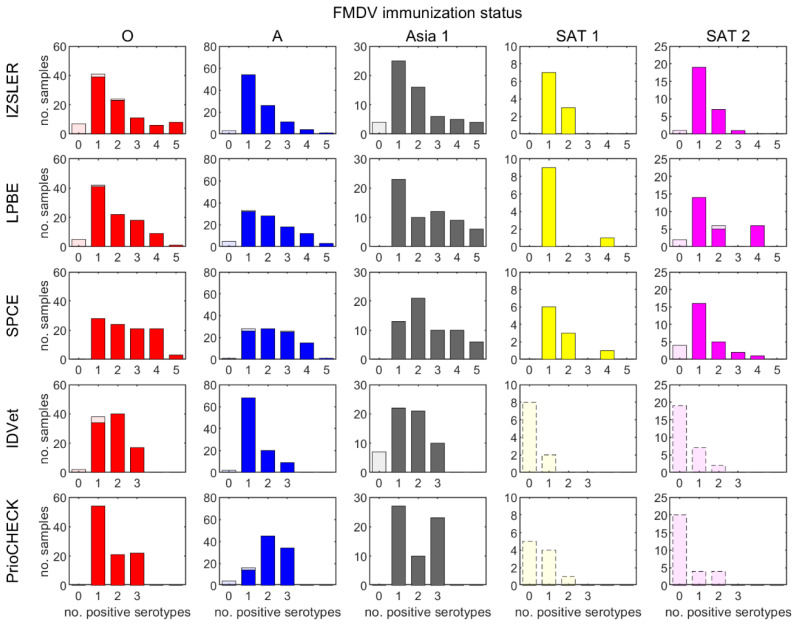
Number of serotypes for which each serum sample from an animal immunized with FMDV serotypes O (red), A (blue), Asia 1 (grey), SAT 1 (yellow) or SAT 2 (magenta) (indicated above the top panel) were positive (based on the specified spot test dilutions and cut-offs) when tested using an ELISA against FMDV serotype O, A, Asia 1, SAT 1 or SAT 2. Results are shown for five SP-ELISAs: IZSLER, liquid-phase blocking ELISA (LPBE), solid-phase competition ELISA (SPCE), IDVet and PrioCHECK™. Dark and pale colors show counts for samples that were positive and negative, respectively, for the homologous serotype. However, for IDVet and PrioCHECK™, tests were not available against serotypes SAT 1 or SAT 2; therefore, only heterologous reactivity is shown (indicated by the dashed border for the bars).

Using a spot test format (with the serum dilution recommended in each of the kits), most sera scored positive for the homologous serotype regardless of the test system used (Figure 1). The diagnostic sensitivity for serotypes O, A and Asia 1 (Table 1) ranged from 88% (the IDVet test for serotype Asia 1) to 100% (the PrioCheck and SPCE tests for serotypes O and Asia 1 and the LPBE test for serotype Asia 1). Scoring inconclusive results as positive improved the sensitivity in some cases. Most samples that were negative using a test for the homologous serotype produced negative results when tested against four heterologous serotypes, though a small number (*n* = 3 for IZSLER, LPBE and SPCE and *n* = 15 for IDVet and PrioCHECK; Figure 2) were positive for at least one heterologous serotype. From a minimum of 8% (SPCE type A) to a maximum of 92% (LPBE type Asia 1) of sera still scored homologous positive at the final dilution tested in each kit.

### 3.2. Heterologous Testing

All the tests produced cross-reactive responses to serotype(s) that the animals had not been exposed to (Figure 1, Table 1). The association between homologous and heterologous reactivity varied between tests and sera (Table 1, Supplementary Materials Figures S1 and S2). However, regardless of the test system, over half of the samples that were positive using a test for the immunizing serotype (homologous response) were also positive for at least one other serotype (heterologous response) and, in some cases, for all serotypes that were tested (Figure 2). These cross-reactive responses were higher than the set cut-offs (i.e., the samples would have been considered positive) and sometimes higher than the responses measured against the immunizing serotype. However, for each of the five ELISA systems, most sera scored positive at a higher dilution and/or with the highest competition value at a given dilution when the serotype of the ELISA matched the serotype with which an animal had been infected and/or vaccinated. Table 1 shows the results for different comparative interpretations of this type for sera and tests for serotypes O, A and Asia 1. The best specificity was achieved by comparing the competition values at either the spot test dilution or the highest dilution with a positive reaction. In this way, a serotype specificity greater than 90% could be achieved with at least one approach, except with the Priocheck and SPCE type A tests.

### 3.3. Statistical Analysis of the Test Responses

Based on the deviance information criterion (DIC), the responses of the sera for all five ELISAs were best captured using a model that incorporated the serotype, immunization status and species (Supplementary Materials Table S4). There was no evidence that either the length of sample storage or the time at which the serum was collected after immunization influenced the response for any of the ELISAs (Supplementary Materials Table S4).

The homologous responses of all five SP-ELISAs with sera from sheep were significantly lower than those with cattle sera (Table 2). However, there was no consistent difference between the responses for sera from pigs compared with cattle (Table 2), reflecting the small number of porcine samples in the study (Supplementary Materials Table S1). For all five ELISAs, there was no significant difference in the responses for sera from vaccinated animals compared with infected animals, but the responses were significantly higher for sera collected from vaccinated and infected animals compared with those from animals that were either vaccinated or infected (Table 2).

The responses against different serotypes in a serum sample were positively correlated amongst all serotypes for IZSLER, LPBE, IDVet and PrioCHECK (Supplementary Materials Table S5). The correlations were mostly in the range of 0.2 to 0.4, and the highest was 0.6 between O and SAT-1 for LPBE. The correlations between the responses were weaker for SPCE, with some close to zero or even negative (Supplementary Materials Table S5).

**Figure 3:**
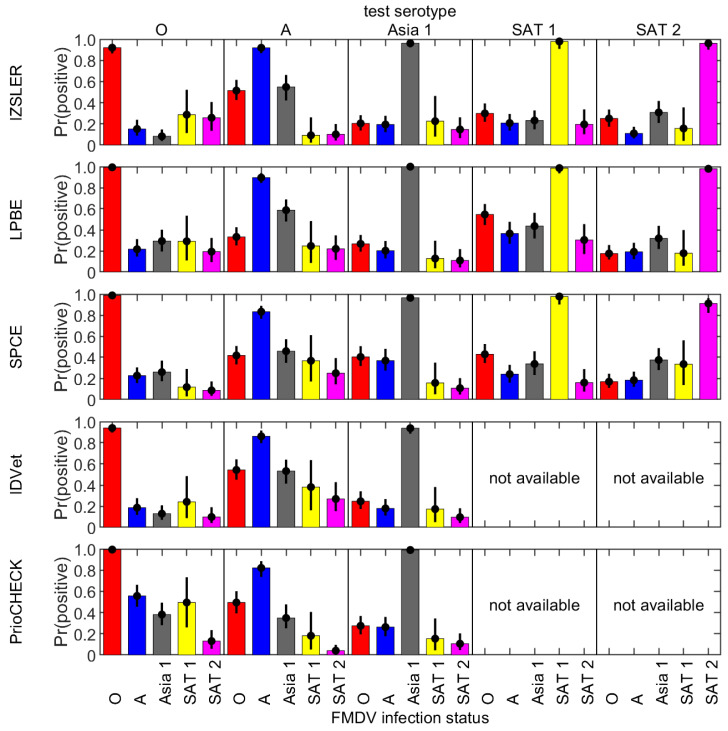
Estimated probability of a positive result when a serum sample from a bovine animal infected with FMDV serotype O (red), A (blue), Asia 1 (grey), SAT 1 (yellow) or SAT 2 (magenta) (indicated below the bottom panel) was tested using an ELISA against FMDV serotype O, A, Asia 1, SAT 1 or SAT 2 (indicated above the top panel). Results are shown for spot tests with five SP-ELISAs: IZSLER, liquid-phase blocking ELISA (LPBE), solid-phase competition ELISA (SPCE), IDVet and PrioCHECK™. Bars and circles indicate the median and the error bars indicate the 2.5th and 97.5th percentiles for the posterior distribution.

The fitted models described above were used to estimate the probability that a sample from an immunized bovine animal will generate a positive result when tested with a serotype-specific ELISA either against the serotype of the sample or any other serotype (Figure 3). Samples were most likely to be positive when tested with an ELISA against the homologous serotype. However, there was still an appreciable probability (>10%) that a sample was positive when tested with an ELISA against a different serotype and, in some cases, this exceeded 50%. The pattern of cross-serotype reactivity varied between tests and serotypes. For example, type A sera cross-reacted more in the SPCE and Priocheck tests than in the other tests (Figure 1, Figure 2 and Figure 3, Table 1).

**Figure 4:**
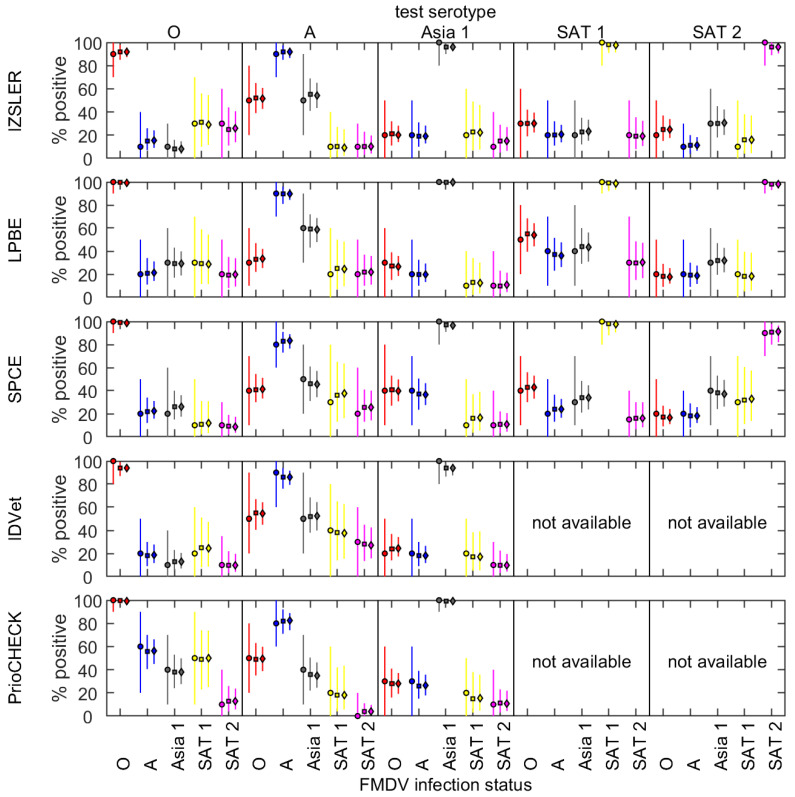
Simulated percentage of positive samples when sera from 10, 100 or 1000 cattle infected with FMDV serotype O (red), A (blue), Asia 1 (grey), SAT 1 (yellow) or SAT 2 (magenta) (indicated below the bottom panel) were tested using an ELISA against FMDV serotype O, A, Asia 1, SAT 1 or SAT 2 (indicated above the top panel). The results are shown for spot tests with five SP-ELISAs: IZSLER, liquid-phase blocking ELISA (LPBE), solid-phase competition ELISA (SPCE), IDVet and PrioCHECK™. Bars and symbols indicate the median and the error bars indicate the 2.5th and 97.5th percentiles for the posterior distribution. The symbols indicate the number of cattle tested: 10 (circles), 100 (squares) or 1000 (diamonds).

The likely proportion of correctly serotyped samples can be simulated using the fitted models assuming only one serotype was in circulation or used for vaccination. This can provide a cut-off for serotype inference when only one serotype is tested for. For example, if 100 or 1000 samples were tested at the spot test dilution from a population exposed to a single FMDV serotype using an ELISA against serotype O and >60% of samples were positive, this would be strongly indicative that serotype O was circulating in the population (Figure 4). Similar results were obtained for serotype Asia 1, but the serotype discrimination was less efficient with tests for serotype A as for the comparative test interpretation (Table 1).

### 3.4. Stabilized Antigens

**Figure 5:**
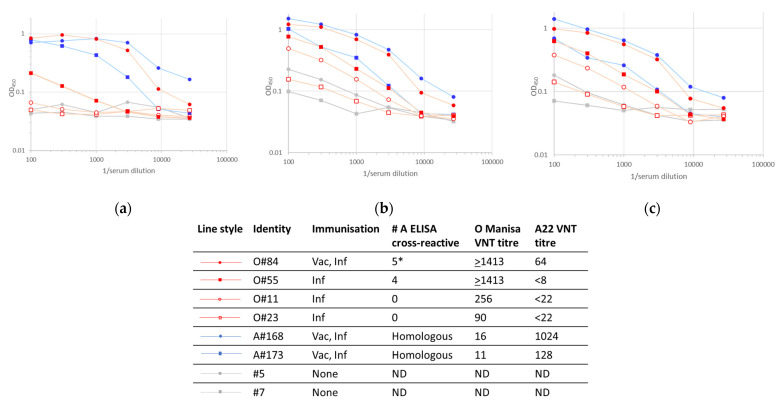
Seroreactivity in indirect ELISAs with A22 capsid antigens captured using recombinant integrin: (**a**) recombinant stabilized capsid antigen; (**b**) wild-type capsid antigen; (**c**) wild-type capsid antigen heated to 60 °C for 10 min. * Number of SP ELISAs in which the serotype A sera scored O positive.

**Figure 6:**
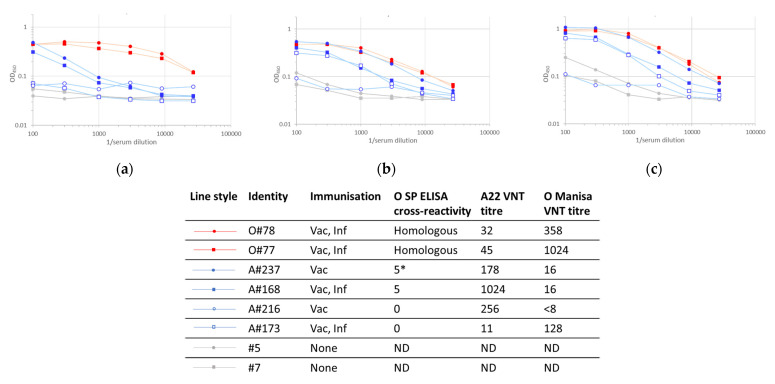
Seroreactivity in indirect ELISAs with O Manisa capsid antigens captured using recombinant integrin: (**a**) recombinant stabilized capsid antigen; (**b**) wild-type capsid antigen; (**c**) wild-type capsid antigen heated to 60 °C for 10 min. * Number of SP ELISAs in which the serotype A sera scored O positive.

**Figure 7:**
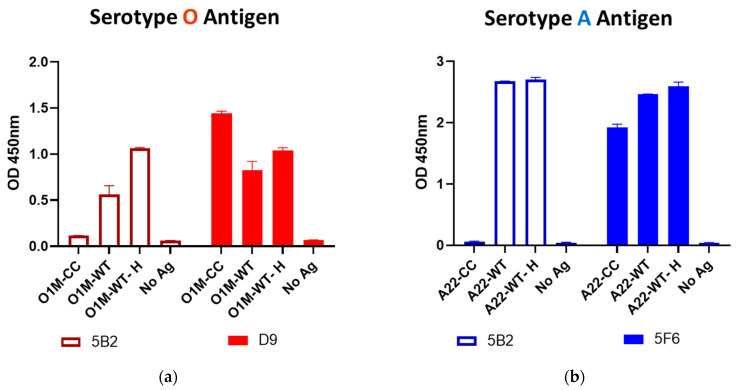
Assessment of serotype O (**a**) and serotype A (**b**) antigen integrity using mAbs directed at an internal epitope on VP2 (5B2) and at surface-exposed neutralizing epitopes (D9 for serotype O, binding to the G-H loop on VP1, and 5F6 for serotype A, identifying a conformational epitope). O1M, antigen prepared from the O Manisa virus; A22, antigen prepared from the A22 Iraq virus; -CC, covalent cage stabilized capsids; -WT, wild-type capsids; -H, heated capsids; No Ag, control without antigen.

To understand the factors that underpin the inter-serotypic cross-reactivity that was observed for the SP-ELISAs, a model system using intact and degraded FMDV capsids was implemented. These assays compared the responses of sera to stabilized, wild-type and heat-treated serotype O and A antigens (Figure 5 and Figure 6), where the integrities of the different antigen preparations were confirmed by the binding of characterized mAbs (Figure 7). These validation experiments showed that VP2-specific internal epitopes (mAb 5B2) were not accessible with the recombinant stabilized capsid antigens, whereas both internal and external epitopes were bound in wild-type antigens and their relative exposures were either unchanged (serotype A) or increased (serotype O internal antigens) after heat treatment, probably depending on the initial integrity status of the capsids. The use of the recombinant stabilized capsids in the integrin-capture ELISA reduced the cross-serotypic reactivity of some, but not all, of the sera tested (Figure 5 and Figure 6). For the serotype A antigen, one serum (O84) from an animal vaccinated and infected with serotype O remained strongly reactive with stabilized capsid.

### 3.5. VNT

**Figure 8:**
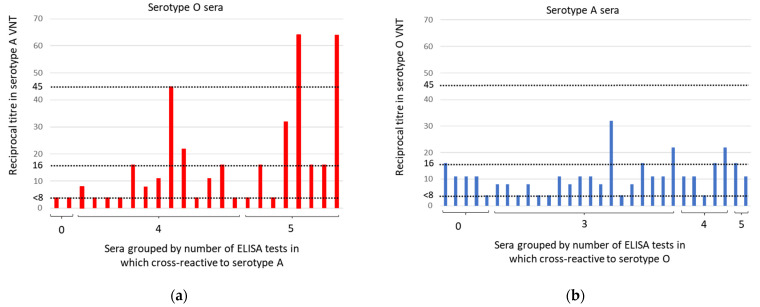
Heterologous VNT results for 53 sera from cattle immunized with serotype O (**a**) or serotype A (**b**). Titres of less than 1 in 8 are shown as 1 in 4. Titres of >1 in 45 were considered positive. Titres of 1 in 16 to 1 in 32 were considered inconclusive.

The reciprocal VNT titers to the homologous serotypes ranged from 22 to 1024 (serotype A) and 90 to >1413 (serotype O). Heterologous VNT serotype results are shown in Figure 8 (excluding seven type O sera showing cytotoxicity). The highest heterologous titers were observed for antisera to serotype O, which were collected after vaccination and a subsequent challenge with the O Manisa virus. One serum that was cross-reactive in four heterologous serotype ELISAs neutralized serotype A at 1 in 45. Two sera (one of which was O84) that were cross-reactive in five heterologous serotype ELISAs neutralized serotype A at 1 in 64. The most cross-reactive serotype A serum, with a heterologous titer to serotype O of 1 in 32, was also derived from an animal after vaccination and challenge, whilst two sera with titers to serotype O of 1 in 22 were collected at 21–56 days after vaccination without challenge. These three serotype A sera were cross-reactive in three or four heterologous serotype SP-ELISAs.

## 4. Discussion

This comparison of FMDV SP-ELISAs confirmed that, as reported for the LPBE [12], the currently used tests were all incompletely serotype-specific. Indeed, approximately half of all positive sera scored positive for at least one serotype other than the immunizing serotype and cross-reactions were detected for antibodies derived from infection, vaccination and vaccination followed by infection. Although in most cases, the strongest reaction was measured for the homologous serotype, our findings have practical relevance for serotyping FMD outbreaks using serological methods. In view of the inter-serotype cross-reactivity that was observed, correct identification of the serotype that animals have been exposed to is difficult unless a single serotype is involved (such as an incursion of the virus into an FMD-free country), and also requires that laboratories use the full serotype range of SP-ELISAs. A common approach that is adopted in serosurveys to detect and quantify undisclosed infection with FMDV in unvaccinated animal populations is to screen samples with an NSP-ELISA and then retest positive samples using SP-ELISA. If SP testing is only used to confirm that infection has occurred, then cross-serotype reactions do not matter, especially if prior knowledge is used to select a single SP-ELISA that matches the prevailing serotype.

In FMD-endemic settings where more than one serotype is present, serotyping is useful to determine which serotype(s) is or was circulating and the protective levels of immunity in susceptible animals. However, the data presented in this study showed that extreme caution is needed when using serological assays to dissect prior exposure of animals to multiple FMD serotypes. If serotyping is required in regions where more than one serotype may have circulated, anti-SP reactivity in more than one SP test could indicate either cross-reactivity or dual infection/vaccination. Considering that background levels of cross-reactivity vary between tests and will be influenced by different antigenic relationships of test and field viruses, distinguishing between these possibilities will be difficult [24,25,26], although there are opportunities to improve the precision of these inferences by analyzing multiple sera using different test formats. To reduce the likelihood of sampling animals exposed to multiple serotypes, surveys should target unvaccinated animals at 6–12 months (less time to become multiply infected and old enough to lose maternal antibodies) and be geographically stratified. This will also provide the most up-to-date and geospatially explicit information on the circulating field virus(es). Casey-Bryars et al. 2018 [27] used modeling informed by outbreak data to infer serotyping from changes in SP reactivity patterns over time, but this involved 19 samplings over three years.

Where SP testing is used to measure immunity in vaccinated animals [28], an antibody response to a polyvalent vaccine might be incorrectly attributed to a vaccine component of a particular serotype when it was a cross-reaction from another component of a different serotype. For example, in the LPBE, an antibody titer around 1 in 100 may approximate the threshold of adequate protection for many vaccines [13,29], and at the 1 in 90 serum dilution used in this study, 10 out of 37 animals vaccinated against serotype A were positive for serotype O.

Antibodies responsible for cross-serotype reactions must bind to conserved capsid epitopes. The perceptions that serotypes do not cross-protect [30] and that vaccines with degraded capsids protect poorly [8] are consistent with the view that most or at least the dominant external capsid epitopes are serotype-specific and that conserved internal epitopes do not elicit protective antibodies. In a preliminary study of the nature of the cross-reactivity between serotypes, a smaller set of serotype O and A antisera were analyzed for binding to stabilized and dissociated ELISA antigens that allow for a distinction to be made between the contribution of internal and external capsid epitopes. Covalently stabilized capsids for serotypes O and A did not present internal capsid epitopes in ELISA where cross-serotype reactivity was reduced, providing evidence that internal epitopes are not serotype-specific. However, some inter-serotypic cross-reactivity remained with the stabilized capsids, pointing to the existence of immunogenic capsid surface epitopes that are conserved between serotypes. These results are consistent with the occurrence of limited cross-serotype neutralization [5,11] and recent studies to identify the epitopes involved [31].

The routinely used SP tests studied here employ cell-culture-derived and chemically inactivated capsid antigens and measure the competition between serum antibodies and the mAb or polyclonal indicator antibodies supplied in the tests. If these antibody reagents bind to serotype-specific external capsid epitopes, this might be expected to make the tests serotype-specific regardless of any degradation of the test antigen. However, for the SPCE and LPBE, the polyclonal test antibodies are themselves prepared via hyperimmunization of laboratory animals with culture-derived and inactivated FMDV antigens [32], some of which may have degraded, giving rise to antibodies directed at internal capsid epitopes. Other tests used in this study employ FMDV-specific mAbs, where serotype specificity can be anticipated to arise from the binding of these reagents to external capsid epitopes. mAb binding data was used to confirm that antigens employed in one commercial kit (IZSLER) presented a mixture of internal and external capsid epitopes (data not shown). We hypothesized that steric interference caused by antibodies in the test sera binding to other epitopes exposed to degraded capsids generated cross-serotypic signals in SP-ELISAs. However, we could not demonstrate this effect using the mAb 5B2 that binds to internal capsid epitopes, as it did not significantly inhibit the binding of the IZSLER test conjugates for serotypes O or A (data not shown). Nevertheless, the broader range of epitopes that are expected to be bound by the polyclonal antibodies in the sera of immunized animals might do so.

Evidence for an internal capsid antigen that reacts with both serotype-specific (homotypic) and heterotypic antibodies and is a degradation product of capsids that have homotypic specificity has been available for many years [33]. Using complement fixation tests, these authors showed that cross-reactivity of capsids increased after storage at 4 °C for one week. The importance of avoiding antigen degradation is well known for FMD vaccines that may be purified using ultracentrifugation, must not be frozen and thawed, and have a limited shelf-life. Similar precautions should be taken by those making and using FMD SP-ELISAs and the antigens are generally purified prior to use or else “on the plate” using capture antibodies. The procedure for virus inactivation is often a critical step in antigen preparation as it cannot be done at refrigeration temperatures and may need to be carried out twice for safety reasons. Freezing and thawing antigens should be avoided and cryoprotectants can stabilize antigens for storage at sub-zero temperatures [34,35,36].

## 5. Conclusions

In summary, this paper documents a significant degree of serotype cross-reactivity exhibited by all SP-ELISAs that are used for FMDV serology. Therefore, extreme caution must be taken in using them for serotyping. No test format was obviously superior overall in terms of sensitivity and specificity, although some serotype A tests were particularly cross-reactive. The patterns of cross-reactions were not test-independent and it is likely that specificity differences were influenced by the priority given to sensitivity versus specificity (reflected in the chosen cut-off) via the use of different strains to incorporate in vaccines and to prepare antigens for different tests of a given serotype, as well as the binding characteristics of competitor antibody reagents, including unique mAbs used in some tests. We provided data to support the idea that cross-serotype reactive responses were due in part to the binding of test sera to internal capsid epitopes, although the reactivity of some sera indicated that external capsid epitopes may also contribute. Degraded vaccines gave rise to cross-reactive antibodies that fail to provide good protection [8,9], but that may be especially cross-reactive in ELISAs. This can cause problems in the use of SP-ELISAs as indicators of protection in vaccinated animals and may account for the poor correlation that is sometimes seen between SP-ELISAs and VNT, not only in estimates of protection but also cross-protection [37]. These findings suggest that VNT may be a more reliable indicator of protection and cross-protection and provide impetus to work to identify serotype-specific epitopes that might be accommodated in new SP-ELISAs.

## Figures and Tables

**Table 1:** Sensitivity and specificity of ELISAs for FMDV serotypes O, A and Asia 1 with sera raised against these three serotypes (inconclusive positive results scored negative).

Sensitivity (Se)/Specificity (Sp) for Homologous Test	Type O Sera Tested with Kits for Types O, A and Asia 1					Type A Sera Tested with Kits for Types O, A and Asia 1					Type Asia 1 Sera Tested with Kits for Types O, A and Asia 1				
	IZSLER	PRIOCHECK	IDVET	SPCE	LPBE	IZSLER	PRIOCHECK	IDVET	SPCE	LPBE	IZSLER	PRIOCHECK	IDVET	SPCE	LPBE
Se (spot)	0.90	1.00	0.94	1.00	0.94	0.97	0.94	0.98	0.96	0.94	0.93	1.00	0.88	1.00	1.00
Sp1 (spot)	0.49	0.56	0.37	0.40	0.63	0.72	0.15	0.70	0.37	0.66	0.55	0.45	0.42	0.57	0.47
Sp2 (spot)	**0.94**	**0.99**	0.88	**0.99**	**0.99**	**1.00**	0.57	0.99	0.87	**0.99**	**0.96**	0.93	**0.94**	**0.95**	0.80
Sp1 (titration)	0.73	0.97	0.91	0.91	0.84	1.00	0.68	0.92	**0.97**	0.88	0.75	0.95	0.74	0.84	0.66
Sp2 (titration)	0.92	**0.99**	**0.98**	0.97	0.97	**1.00**	**0.78**	**1.00**	**0.97**	0.96	0.95	**0.97**	0.93	0.92	**0.95**

Bold text shows the highest specificity; Se (spot)—proportion of sera scored positive in homologous spot test; Sp1 (spot)—proportion of sera scored positive in the homologous spot test that were not positive in the heterologous spot tests; Sp2 (spot)—proportion of positive sera scored most strongly positive in the homologous spot test; Sp1 (titration)—proportion of positive sera that were only homologous positive at the highest positive dilution; Sp2 (titration)—proportion of positive sera scoring as most strongly positive to homologous serotype at the highest test positive dilution.

**Table 2:** Effect of immunization status and species on differences in mean response (percent inhibition or S/N ratio) * between animals for five serological ELISAs for FMDV.

	IZSLER	LPBE	SPCE	IDVet ^†^	PrioCheck
Immunization status (*β_i_*)					
Infected	Baseline	Baseline	Baseline	Baseline	Baseline
Vaccinated	−0.07	−0.02	0.01	−0.01	−0.01
	(−0.31, 0.16)	(−0.27, 0.22)	(−0.17, 0.19)	(−0.23, 0.21)	(−0.23, 0.21)
Vaccinated and infected	0.65	0.48	0.57	−0.72	0.89
	(0.40, 0.88)	(0.23, 0.71)	(0.39, 0.74)	(−0.93, −0.51)	(0.67, 1.10)
Species (*γ_s_*)					
Bovine	Baseline	Baseline	Baseline	Baseline	Baseline
Ovine	−0.92	−0.85	−0.19	0.82	−0.68
	(−1.24, −0.59)	(−1.19, −0.50)	(−0.43, 0.06)	(0.51, 1.14)	(−1.00, −0.38)
Porcine	0.004	−0.18	−0.33	−1.34	−0.06
	(−0.58, 0.59)	(−0.78, 0.41)	(−0.76, 0.09)	(−1.90, −0.79)	(−0.59, 0.47)

* Posterior median (95% credible interval); ^†^ note: the response for the IDVet ELISA (S/N ratio) is on the opposite scale to that for the other four tests (percent inhibition).

## Data Availability

Not applicable.

## Supplementary Materials

The following supporting information can be downloaded at https://www.mdpi.com/article/10.3390/v14071495/s1. Figure S1: Comparison of homologous and heterologous serotypic responses for sera from animals immunized with FMDV when tested using an ELISA. Figure S2: Comparison of homologous response and number of heterologous serotypes for which a sample was positive for sera from animals immunized with FMDV. Table S1: Characteristics of sera. Table S2: Serological assays used for cross-reactivity study. Table S3: Serum dilutions and cut-offs for SP-ELISAs. Table S4: Deviance information criterion for models incorporating different factors influencing the mean response of five serological ELISAs for foot-and-mouth disease virus. Table S5: Estimates for the standard deviations and correlation coefficients in the models for the transformed response when sera were tested using SP-ELISAs against different FMDV serotypes.
