# Supplementary material for: Cross-Serotype Reactivity of ELISAs Used to Detect Antibodies to the Structural Proteins of Foot-and-Mouth Disease Virus
Source: Viruses. 2022 Jul 8;14(7):1495. doi: 10.3390/v14071495 (PMC9316314; doi:10.3390/v14071495)
Supplement: Supplementary file 1 [file viruses-14-01495-s001.zip › Supplementary Figures.pdf]

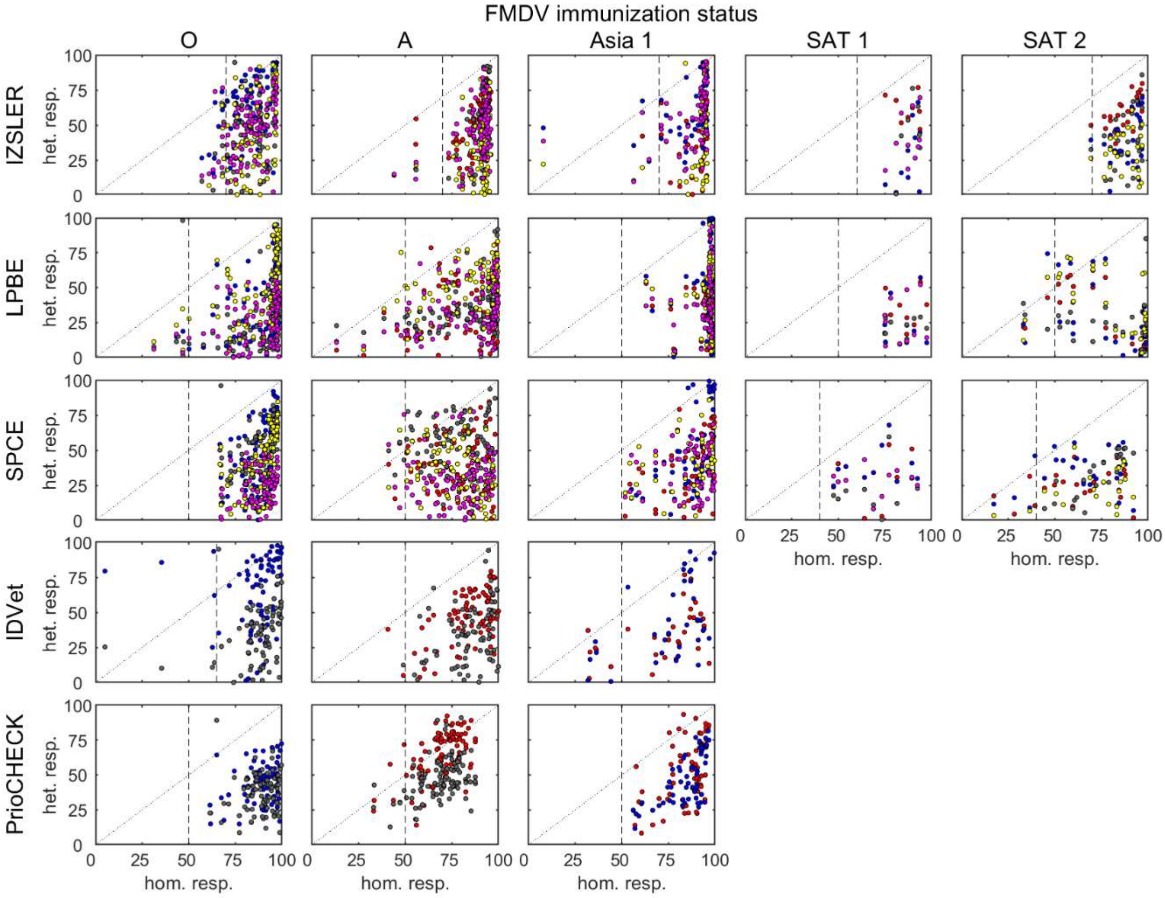

Figure S1: Comparison of homologous and heterologous serotypic responses for sera from animals immunized with FMDV serotype O, A, Asia 1, SAT 1 or SAT 2 (indicated above the top panel) when tested using an ELISA against FMDV serotype O (red), A (blue), Asia 1 (grey), SAT 1 (yellow) or SAT 2 (magenta). Results are shown for five ELISAs: IZSLER, liquid-phase blocking ELISA (LPBE), solid-phase competition ELISA (SPCE), IDVet and PrioCHECK™. The dashed lines indicate the homologous spot test cut-off and the dotted lines indicate equality of response (i.e., for points above the dotted line, the heterologous response was greater than the homologous one).

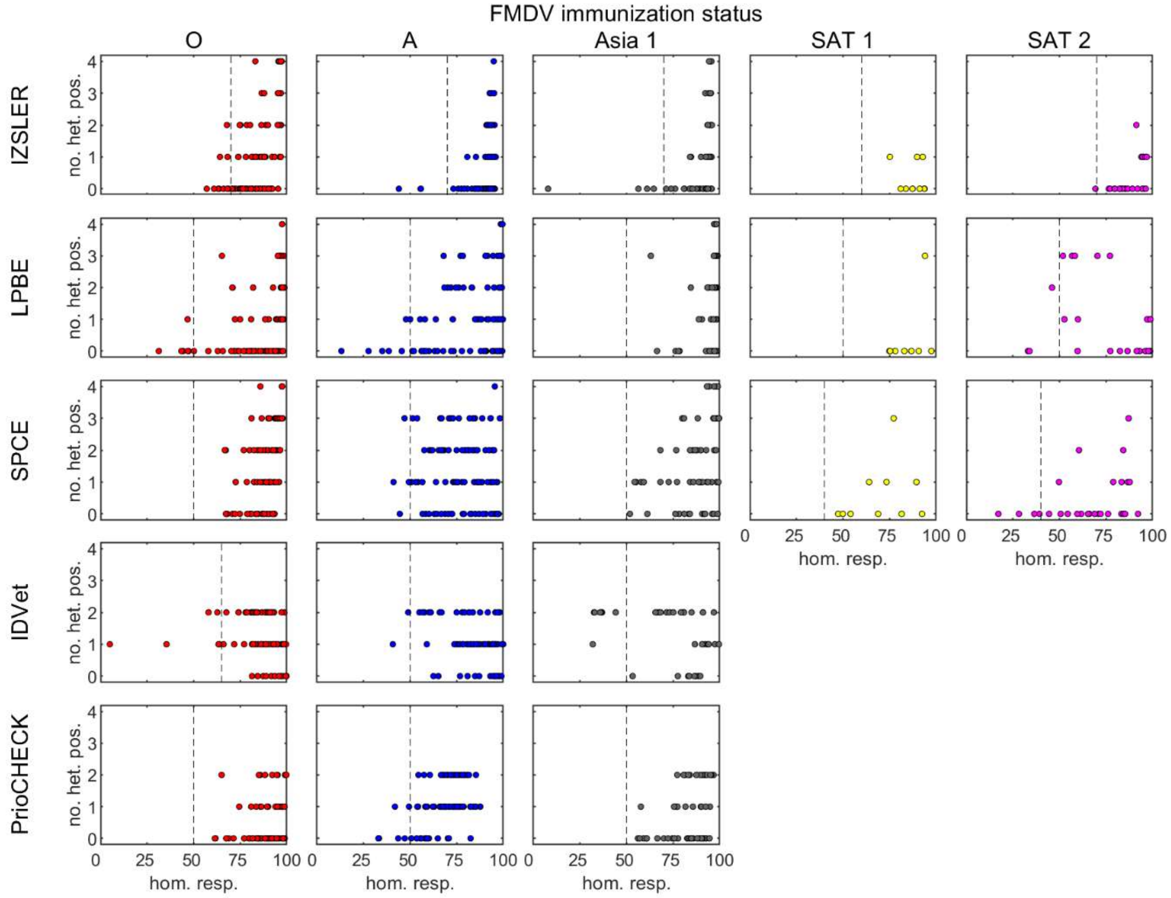

Figure S2: Comparison of homologous response and number of heterologous serotypes for which a sample was positive for sera from animals immunized with FMDV serotype O (red), A (blue), Asia 1 (grey), SAT 1 (yellow) or SAT 2 (magenta) (indicated above the top panel) when tested using an ELISA against heterologous FMDV serotypes. Results are shown for five ELISAs: IZSLER, liquid-phase blocking ELISA (LPBE), solid-phase competition ELISA (SPCE), IDVet and PrioCHECK™. The dashed line indicates the cut-off for the homologous spot test.
