# Supplementary material for: Cross-Serotype Reactivity of ELISAs Used to Detect Antibodies to the Structural Proteins of Foot-and-Mouth Disease Virus
Source: Viruses. 2022 Jul 8;14(7):1495. doi: 10.3390/v14071495 (PMC9316314; doi:10.3390/v14071495)
Supplement: Supplementary file 1 [file viruses-14-01495-s001.zip › Supplementary tables.pdf]

Cross-serotype reactivity of ELISAs detecting antibodies to the structural proteins of foot-and-mouth disease virus

Supplementary Tables

Table S1. Characteristics of sera.

characteristic	immunizing serotype					all
	O	A	Asia 1	SAT 1	SAT 2	
species*						
bovine	69	99	55	10	28	261
ovine	21	0	5	0	0	26
porcine	7	0	0	0	0	7
immunization status*						
infected	56	13	20	1	4	94
vaccinated	21	35	17	9	12	94
infected/vaccinated	20	51	23	0	12	106
time sample taken†	35 (8-431)	32 (13-62)	33 (7-343)	21 (21-21)	36 (11-58)	32 (7-431)
age of sample‡	11.6 (2.4-48.2)	13.0 (3.0-43.3)	11.1 (3.0-17.4)	11.7 (7.4-56.0)	12.7 (2.8-46.3)	12.7 (2.4-56.0)
total no. samples*	97	99	60	10	28	294

* number of samples

† median (range) days post infection or vaccination when sample taken

‡ median (range) age of sample in years

Table S2. Serological assays used for cross-reactivity study

ELISA type*	Description	Type	Ligand type	Serotypes tested
Liquid-phase blocking ELISA	In-house LPBE	In-house	Polyclonal	O, A, Asia 1, SAT 1 and SAT 2
Solid-phase competition ELISA	In-house SPCE	Commercial	Monoclonal	O, A, Asia 1, SAT 1 and SAT 2
	IZSLER SPCE	Commercial	Monoclonal	O, A, Asia 1, SAT 1 and SAT 2
	ID Screen® FMD	Commercial	Monoclonal	O, A and Asia 1
	PrioCHECK™	Commercial	Monoclonal	O, A and Asia 1
Indirect integrin ELISA	Bespoke	In-house	Integrin/Monoclonal	O and A
VNT	WRLFMD	In-house	Not applicable	O and A

*All use cell culture derived FMDV antigens, except the integrin ELISA which used recombinant FMDV capsids with or without stabilizing mutations

Table S3. Serum dilutions and cut-offs for SP-ELISAs

Test	Reciprocal dilutions analyzed*				Cut-off
LPBE	10	30	<u>90</u>	270	≥50%
SPCE O, A, Asia 1	<u>10</u>	30	90	270	≥50%
SPCE SAT 1, SAT 2	<u>10</u>	30	90	270	≥40%

IZSLER O, A, Asia 1, SAT 2	10	30	90	270	≥70%
IZSLER SAT 1	10	30	90	270	≥60%
PrioCHECK O	10	30	90	270	≥50%
PrioCHECK A/Asia 1	5	15	45	135	≥50%
IDVet	3.5	10.5	31.5	94.5	≤35%
IDVet O Porcine	3.5	10.5	31.5	94.5	≤50%

* Dilution used for spot test is underlined

Table S4. Deviance information criterion for models incorporating different factors influencing the mean response of five serological SP-ELISAs for FMDV.

model	deviance information criterion (DIC)*				
	IZSLER	LPBE	SPCE	IDVet	PrioCheck
serotype+immunization status+species+time sample taken+age of sample	4269	4242	3990	2406	2298
serotype+immunization status+species+time sample taken	4268	4240	3988	2406	2298
serotype+immunization status+species +age of sample	4270	4242	3992	2407	2296
serotype+immunization status+species	4268	4240	3990	2407	2296
serotype+immunization status	4294	4259	3990	2457	2311
serotype+ species	4315	4261	4045	2468	2382
serotype	4350	4289	4051	2523	2408
intercept only	5401	5653	4924	3196	3137

* a model with a lower DIC is preferred to one with higher DIC; however, if the difference between models is less than two, the simpler model is preferred as it has the smaller number of parameters

Table S5. Estimates* for the standard deviations and correlation coefficients in the models for the transformed response when sera are tested using ELISAs against different foot-and-mouth disease virus serotypes

test serotype(s)	IZSLER	LPBE	SPCE	IDVet	PrioCheck
standard deviation					
O	0.97 (0.89, 1.05)	1.04 (0.96, 1.13)	1.04 (0.96, 1.13)	0.91 (0.84, 0.99)	0.97 (0.90, 1.06)
A	1.21 (1.11, 1.32)	1.57 (1.45, 1.71)	1.15 (1.06, 1.25)	1.05 (0.96, 1.14)	0.91 (0.83, 0.99)
Asia 1	1.15 (1.06, 1.25)	1.07 (0.99, 1.17)	0.89 (0.82, 0.97)	0.91 (0.84, 0.99)	0.86 (0.80, 0.94)
SAT 1	1.19 (1.10, 1.30)	0.95 (0.88, 1.04)	0.87 (0.80, 0.94)	-	-
SAT 2	0.96 (0.89, 1.05)	1.02 (0.95, 1.12)	0.89 (0.82, 0.97)	-	-
correlation coefficient					
O/A	0.45 (0.35, 0.54)	0.31 (0.20, 0.41)	0.31 (0.20, 0.41)	0.16 (0.05, 0.28)	0.34 (0.23, 0.43)
O/Asia 1	0.27 (0.16, 0.38)	0.31 (0.20, 0.41)	0.22 (0.11, 0.34)	0.20 (0.10, 0.31)	0.30 (0.19, 0.40)
O/SAT 1	0.24 (0.13, 0.35)	0.61 (0.53, 0.68)	0.36 (0.25, 0.45)	-	-
O/SAT 2	0.21 (0.10, 0.32)	0.26 (0.14, 0.36)	0.01 (-0.11, 0.13)	-	-
A/Asia 1	0.38 (0.28, 0.49)	0.30 (0.19, 0.40)	0.25 (0.14, 0.35)	0.30 (0.19, 0.40)	0.31 (0.20, 0.41)
A/SAT 1	0.34 (0.23, 0.44)	0.38 (0.27, 0.47)	0.12 (0.00, 0.23)	-	-
A/SAT 2	0.29 (0.17, 0.39)	0.21 (0.10, 0.31)	0.12 (0.00, 0.23)	-	-
Asia 1/SAT 1	0.33 (0.22, 0.42)	0.43 (0.33, 0.52)	0.35 (0.24, 0.45)	-	-
Asia 1/SAT 2	0.32 (0.21, 0.42)	0.25 (0.13, 0.35)	-0.02 (-0.13, 0.10)	-	-
SAT 1/SAT 2	0.23 (0.12, 0.34)	0.39 (0.28, 0.48)	-0.06 (-0.18, 0.06)	-	-

* posterior median (95% credible interval)
